# Analysis of Essential Oils Components from Aromatic Plants Using Headspace Repellent Method against *Aedes aegypti* Mosquitoes

**DOI:** 10.3390/molecules28114269

**Published:** 2023-05-23

**Authors:** Mohammad Adam Mustapa, Ikhsan Guswenrivo, Ade Zurohtun, Nur Kusaira Khairul Ikram, Muchtaridi Muchtaridi

**Affiliations:** 1 Department of Pharmaceutical Analysis and Medicinal Chemistry, Faculty of Pharmacy, Padjadjaran University, Jatinangor, 45363, Indonesia; mohamad20015@mail.unpad.ac.id; 2 Department of Pharmacy, Faculty of Sports and Health, Gorontalo State University, Gorontalo 96211, Indonesia; 3 Research Center for Applied Zoology, Research Organization for Life Sciences and Environmemt, National Research and Innovation Agency(BRIN), Jakarta 10340, Indonesia; ikhsan.guswenrivo@brin.go.id; 4 Department of Biological Pharmacy, Faculty of Pharmacy, Padjadjaran University, Jatinangor 45363, Indonesia; ade.zuhrotun@unpad.ac.id; 5 Institute of Biological Sciences, Faculty of Science, Universiti Malaya, Kuala Lumpur 50603, Malaysia; nkusaira@um.edu.my; 6 Research Collaboration Centre for Theranostic Radiopharmaceuticals, National Research and Innovation Agency (BRIN), Jakarta 10340, Indonesia

**Keywords:** essential oil, headspace repellent, *Aedes aegypti*

## Abstract

This research serves as the basis for developing essential oil-based repellent activity tests against *Aedes aegypti* mosquitoes. The method used for the isolation of essential oils was the steam distillation method. Virus-free *Aedes aegypti* mosquitoes were used as test animals by applying the 10% essential oil repellent on the arms of volunteers. The analysis of the essential oils activities and aromas’ components was carried out using headspace repellent and GC-MS methods. Based on the results, the yields of essential oil from 5000 g samples for cinnamon bark, clove flowers, patchouli, nutmeg seed, lemongrass, citronella grass, and turmeric rhizome were 1.9%, 16%, 2.2%, 16.8%, 0.9%, 1.4%, and 6.8%, respectively. The activity test showed that the average repellent power of 10% essential oils, patchouli, cinnamon, nutmeg, turmeric, clove flowers, citronella grass, and lemongrass, was 95.2%, 83.8%, 71.4%, 94.7%, 71.4%, 80.4%, and 85%, respectively. Patchouli and cinnamon had the best average repellent power. Meanwhile, the aroma activities showed that the average repellent power of the patchouli oil was 96%, and the cinnamon oil was 94%. From the GC-MS analysis, nine components were identified in the patchouli essential oil aromas’ with the highest concentration being patchouli alcohol (42.7%), Azulene, 1,2,3,5,6,7,8,8a-octahydro-1,4-dimethyl-7-(1-methylethenyl)-, [1S-(1α,7α,8aβ)] (10.8%), α-guaiene (9.22%), and seychellene (8.19%)., whereas using the GC-MS headspace repellent method showed that there were seven components identified in the patchouli essential oil aroma with a high concentration of the components, which were patchouli alcohol (52.5%), Seychellene (5.2%), and α-guaiene (5.2%). The analysis results of cinnamon essential oil using the GC-MS method showed that there were five components identified in the aroma, with E-cinnamaldehyde (73%) being the highest component, whereas using the GC-MS headspace repellent method showed that there were five components identified in the aroma, with highest concentrations of cinnamaldehyde (86.1%). It can be concluded that the chemical compounds contained in patchouli and cinnamon bark have the potential to be environmentally friendly repellents in controlling and preventing *Aedes aegypti* mosquitoes.

## 1. Introduction

Indonesia is rich in natural aromatic materials, consisting of three types of aromatic plants from 22 different families, namely Asteraceae [1], Hypericaceae [2], Lauraceae, Myrtaceae [3], Pinaceae [4], Rubiaceae [5], Rutaceae, and Solanaceae [6], each having major aromatic compound components known as essential oils [7,8].

Essential oil is a secondary metabolite produced by plants that is synthesized through the mevalonic acid pathway [9]. This compound has a distinct smell and vaporizes rapidly; therefore, it is also called volatile oil, ether oil, or essential oil [10]. Essential oils have many health benefits, such as being antioxidant, antimicrobial, antiviral, anticancer, antiprotozoal, cytotoxic, antigenotoxic, antimutagenic, chemopreventive, anti-inflammatory, antinociceptive, immunostimulant, organoprotective, antidiabetic, lipid-lowering, hypoallergenic, antiplatelet, antithrombotic and antitoxin [11]. The compounds in essential oils, when inhaled, also interact with the olfactory system in the central nervous system, stimulating some nerves in the brain [12].

Besides health benefit, essential oil can act as an insecticide. Common insecticides contain toxic chemical compounds such as formaldehyde, pyrethrum, octachlorodipropyl ether, and diethyltoluoamide (DEET), and are thus able to kill all types of insects [13,14]. However, insecticides are under the class of toxic and harmful substances for humans and non-target organisms, and destroy the ozone layer [15]. Insecticides has been widely used as a repellent to control and prevent diseases transmitted by mosquitoes, such as *Aedes aegypti* that causes dengue fever [16]. Therefore, repellents made from natural ingredients could be a safe solution to control *Aedes aegypti* mosquitoes, replacing existing insecticides.

One of the natural repellents derived from aromatic plants that can be developed and diversified into products that are safe for human health as well as the environment is essential oils [15]. Traditionally, essential oils have long been used to repel insects in the form of pests of grains and nuts in storage warehouses [17]. Essential oils can have several effects on insects, namely as repellents [18], attractants [19], contact poisons, fumigants [20], antifeedant [21], and oviposition deterrent [22,23].

Essential oils with activity as repellents are clove *(Syzygium aromaticum* L.) [24], cinnamon (*Cinnamomum burmanii*) [25], nutmeg (*Myristica fragrans*) [26] turmeric (*Curcuma longa* L.) [27], citronella grass (*Cymbopogon nardus*) [28], lemongrass (*Cymbopogon citratus*) [29], and patchouli (*Pogostemon cablin*) [30]. Essential oils from mostly phenylpropanoid and terpenoid compounds can be extracted and isolated from the plant’s parts using steam distillation. Phenylpropanoids are a group of natural phenolic compounds derived from the aromatic amino acids phenylalanine and tyrosine [31]. Terpenoids are isoprene-based natural products that play a fundamental role in the metabolism of all organisms [32]. Terpenoid compounds that can repel insects and other arthropods are monoterpenes and sesquiterpenes [33].

Gas chromatography–mass spectrometry (GC-MS) is the characterization technique widely used to identify components of essential oils by comparing the mass spectrum of the samples with the mass spectrum in the database [34]. Besides GC-MS, another method is headspace (HS) chromatography sampling method. Headspace (HS) is a gas (vapor-phase) chromatography used to determine volatile organic compounds trapped above the matrix in the gas phase. This method is mainly used for taking samples in conditions where the matrix property precludes direct injection into gas chromatography [35]. Headspace gas chromatography ion mobility spectrometry (HS GC-IMS) is a sensitive, fast, and accurate analytical technique used to identify and quantify volatile compounds [36]. HS GC-IMS has been used to characterize and evaluate various products (such as cheese) and other vegetable oils [37].

In this research, a new equipment has been developed, namely the GC-MS headspace repellent. The GC-MS headspace repellent equipment was adopted from the headspace static method [35] to identify the components of essential oils aroma with a pattern of selectivity levels against *Aedes aegypti* mosquitoes. This equipment has been made from materials that are simple, accessible, and cost effective, and can hence be applied in the field. 

## 2. Results

### 2.1. Distillation and Yield of Essential Oils

The essential oil and water vapor evaporate separately during water distillation. Initially, the water evaporated after the heating process, and after reaching a balance at a certain pressure, the water vapor entered the inner tissue of the material and forced the essential oil to the surface. The essential oil will evaporate with the water vapor to the condenser; however, the temperature process of the essential oil is relatively higher than the water vapor [38]. However, steam distillation produced stable steam and heat with a constant vapor pressure. The steam used had a pressure >1 atm (atmosphere) and a temperature >100 °C so that the distillation time is shorter, thus reducing the possibility of damage to the essential oil. The percentage of compounds in the essential oil resulting from steam distillation has a greater value than that from water distillation. The yield percentage of the essential oil was then calculated to determine the amount of dissolved chemical content. 

The results of the distillation with a herbal crude extract weight of 5 kg each showed a difference in the percent yield of essential oils in this case cinnamon bark with a yield of 1.9%, clove flower 16%, patchouli 2.2%, nutmeg 16.8%, lemongrass 0.9%, lemongrass 1.4% and 6.8% turmeric rhizomes.

### 2.2. Bioactivity Test of the Essentials Oils against Aedes aegypti Mosquitoes

In this research, the bioactivity test of the cinnamon bark, clove flowers (eugenol, caryophyllene), patchouli (alpha-guaiene, azulene and patchouli alcohol), nutmeg (beta-phellandrene, cyclohexen, benzodioxole), lemongrass (citral and neral), citronella grass (citronella), and turmeric (turmerone) essential oils as repellents against *Aedes aegypti* adult female mosquitoes was carried out for 6 h. Observations started from hour 0 to hour 6 and were repeated three times. The strain of the mosquitoes used was sterile; therefore, it was safe and free from parasites or virus. The mosquitoes used were female because only female mosquitoes need blood protein for their egg’s maturation process, while male mosquitoes only need nectar or plant extracts. The age of the mosquitoes ranged from 7 to 24 days. The time of testing was morning, from 08.00 a.m. to 2.00 p.m., Western Indonesia Time (WIB). The mosquitoes were active in the surrounding environment of their nesting place in the morning, thus making this the ideal time for testing. During the 6 h treatment, the arm was smeared with essential oil only once at 0 h, and the same arm was used for each consecutive hour. The arm was not washed with the aim to determine the effectiveness of the repellent power produced by each essential oil for 6 h of treatment. The test results of the average repellent power of the essential oils are presented in Table 1.

The test results of the average repellent power of patchouli, cinnamon bark, nutmeg seed, turmeric rhizomes, clove flowers, citronella grass, and lemongrass essential oils are illustrated in Table 1. From the results, it can be concluded that the longer the duration of the treatment, the lower the repellent power of the essential oils. 

### 2.3. Activity Test of the Patchouli and Cinnamon Bark Essential Oils Aroma Using Headspace Repellent Method

The activity test of the patchouli and cinnamon bark essential oils aroma against *Aedes aegypti* using the headspace repellent method were carried out for 5 min and the observation was conducted three times. The results of the patchouli and cinnamon bark essential oils aroma against *Aedes aegypti* mosquitoes using the headspace repellent method are presented in Table 2.

Based on the data presented in Table 2, the average repellent power for patchouli and cinnamon was 96% and 94%, respectively. This indicates that the repellent power of the essentials oils was very effective because the values were above ≥90% [18]. In principle, this headspace repellent method releases compounds through the aroma, which is sensed by the mosquito’s receptors, and proceed to the nerve impulses, causing the mosquito to be dizzy by the unpleasant smell. The mosquito’s brain then responds to avoid the aroma. The repellent variations of patchouli leaves oil and cinnamon bark oil was created based on the first inhalator to the second inhalator for 5 min [39].

### 2.4. Analysis of Essential Oils Aromas’ Components Using GC-MS Method

**Figure 1:**
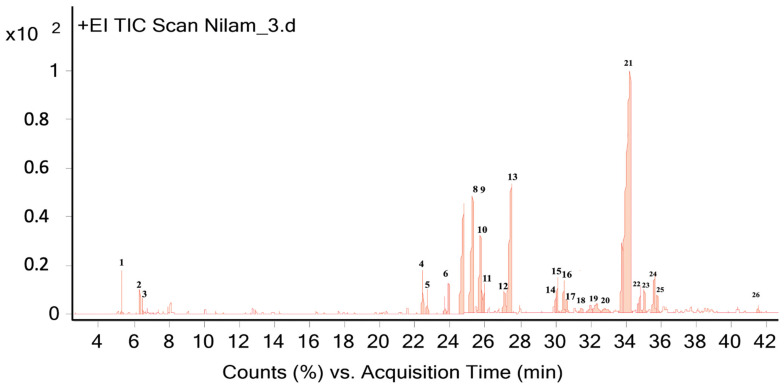
Chromatogram of patchouli essential oil aroma using GC-MS method.

**Figure 2:**
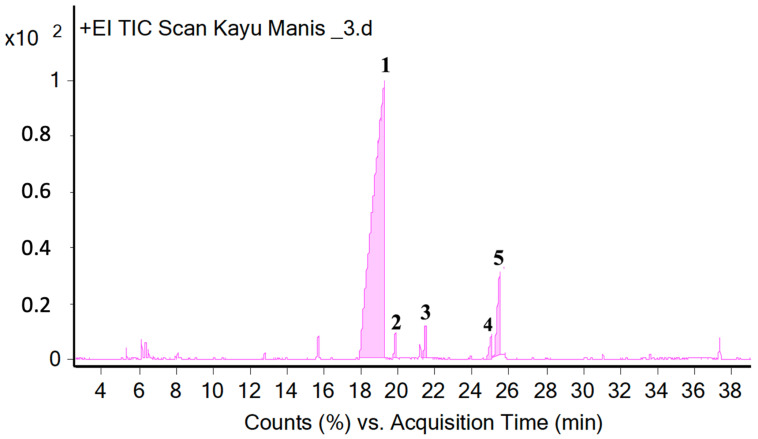
Chromatogram cinnamon bark essential oil using GC-MS.

The aroma of the patchouli and cinnamon bark essential oils obtained from the steam distillation were analyzed using gas chromatography-mass spectrometry (GC-MS). The analysis showed that the aroma of patchouli essential oil contained 26 components as presented in Table 3 and Figure 1. The analysis on cinnamon essential oil contained five components, as presented in Table 4 and Figure 2.

### 2.5. Analysis of Essential Oils Aromas’ Components Using GC-MS Headspace Repellent Method

**Figure 3:**
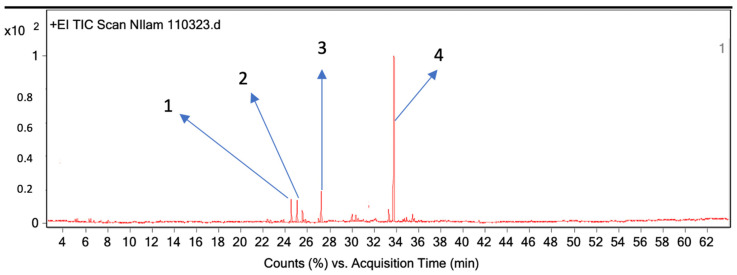
Chromatogram of patchouli essential oil aroma from GC-MS headspace repellent method.

**Figure 4:**
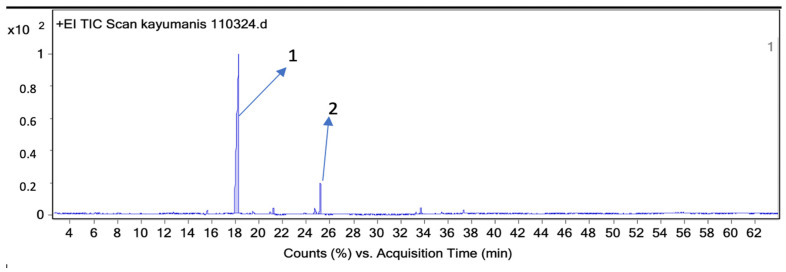
Chromatogram of cinnamon bark essential oil aroma from GC-MS headspace repellent method.

The essential oils of patchouli and cinnamon bark obtained from the steam distillation process were analyzed using gas chromatography–mass spectrometry (GC-MS) combined with the headspace repellent method. The results showed that the aroma of patchouli and cinnamon bark essential oil contained four compounds (Table 5 and Figure 3), and two compounds (Table 6 and Figure 4), respectively. 

## 3. Discussion

The distillation and the yield percentages of essential oils were influenced by several factors, namely moisture content, drying temperature, storage, and the distillation process. Moisture content is one of the important parameters in the production of essential oil. Essential oil is stored in plant tissues and protected by water; therefore, if the water content is too high, it will be difficult for the oil to evaporate during distillation. On contrary, if the water content is too low, the essential oil will evaporate during the drying process [46]. 

Drying is a process of transferring heat and water vapor from the surface of a material using heat energy. It can be carried out by direct sunlight (open sun drying) or by artificial means using tools [47]. In this study, the drying process was carried out to maintain the active components of the plant by reducing the oxidation process and microbial growth [48]. However, a long drying time can reduce the yield percentages of essential oil. Hence, the results showed a decrease in the yield percentage as the temperature increases during the drying process which drastically reduced the production of the essential oils due to high volatility [49]. The choice of drying method is very important for aromatic plants because it can affect the composition of essential oils [50]. Besides, the distillation process also greatly affects the quality of essential oils due to changes from liquid to vapor and vapor to liquid. This distillation process is a method use to separate the components contained in a solution or mixture and depends on the distribution of these components between the vapor phase and the water phase [51].

In addition to the drying process, another factor that affected the content of essential oils was storage conditions. Storage in a room that is not exposed to direct sunlight can cause the loss of essential oils due to oxidation events, while the loss of essential oils due to evaporation is relatively small. Therefore, all materials must be placed in a room where the air is dry, at low temperatures, and free from air circulation [52].

The activity test results of each essentials oil at concentration of 10% against *Aedes aegypti* mosquitoes showed that the average value of the repellent power of patchouli was 95.2%; cinnamon bark, 94.7%; nutmeg seed, 85%; turmeric, 83.8%; clove flowers, 80.4%; citronella grass, 72.2% and lemongrass, 71.4%. These results also showed that the highest average value of essential oils repellent power was patchouli (95.2%) and cinnamon bark (94.7%). Based on the government regulation through the Pesticide Commission of the Ministry of Agriculture in 1995, a substance is said to be effective as a repellent if the result of observation that lasted for 6 h had a repellent power of ≥90% (Table 1 and Figure 1). 

Based on the repellent power of the essential oils, the repellent power of patchouli and cinnamon were the most effective, with more than 90%. Therefore, the essential oils activities of patchouli and cinnamon were further tested using the headspace repellent method and analyzed using the GC-MS method. The results showed that the repellent power of patchouli aroma was 96% and that of cinnamon was 94% (Table 2). The high value of patchouli aroma repellent power is due to the dominated components present in it, which are alpha-guaiene (9.22%), azulene (10.8%), and patchouli alcohol (42.7%), whereas the dominant component in the cinnamon aroma was cinnamaldehyde (73.0%). The repellent power activity of the two aromas is relatively high against *Aedes aegypti* mosquitoes [53]. Repellent compounds are known to have aromas that will interfere with the mosquito receptors. Within the range of the repellant, it will be difficult for the mosquito to find the host. Even if some of the mosquitoes manage to land on the host’s skin, these will eventually fly away.

The results of the patchouli essential oil components using the GC-MS method showed that there were 26 components, consisting of alpha-guaiene (9.22%), azulene (10.8%), and patchouli alcohol (47.2%) (Table 5 and Figure 4), and these components were reported by [54]. Among the four dominant compounds, patchouli alcohol had the highest concentration and produced a distinctive aroma that lasted longer [55]. Patchouli alcohol is a yellow liquid included in the tricyclic sesquiterpene derivative compounds which have benefits in various industries, including pharmaceuticals, cosmetics, food, beverages, cigarettes, repellents, and chemicals [56,57]. Various pharmacological activity have also been reported, including anti-influenza virus [58], anti-inflammatory [59], anti-nociceptive [60], anti-ulcerogenic [61], anti-colitis [62], lung protection [63]. 

The results of cinnamon essential oil using the GC-MS method showed that there were three components identified (Table 4 and Figure 3), with cinnamaldehyde being the highest (90.4%). This component had also been reported by [64]. Of the five compounds identified, only cinnamaldehyde has the highest concentration. This compound is an aromatic aldehyde from the phenylpropanoid group, which is a group of natural phenolic compounds derived from the phenylalanine and tyrosine aromatic amino acids responsible for the aroma of cinnamon essential oil [65] Cinnamaldehyde has been used in various food and beverage industries [66], cigarettes [67], food-flavoring agents, and preservatives with a strong taste [7]. It has pharmacological activities such as anti-diabetic [68], anti-obesity [69], anti-microbial [70] and cardiovascular [71]. In addition to that, fragrance compounds in essential oils derived from plants have been proven to have an influence on locomotor activity [72].

**Figure 5:**
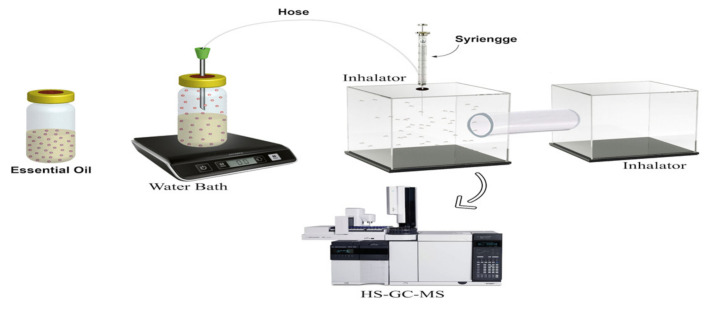
Headspace repellent equipment.

The development of the headspace repellent methods was carried out based on previous research with solvent-free sample preparation where the aroma was taken using a syringe and injected into the GC-MS. The analysis results of the patchouli essential oil aroma using the GC-MS headspace repellent against the *Aedes aegypti* mosquitoes showed that there were four components identified in the aroma. The dominant components were patchouli alcohol (52.5%) with a retention time of 33.77 min, followed by azulene (7.2%) with a retention time of 27.213 min, Seychellene (5.2%) with a retention time of 25.039 min, and alpha-guaiene (5.2%) with a retention time of 24.516 min (Table 5 and Figure 4). Patchouli alcohol as the dominant component in headspace repellent methods has repellency as reported by Zhu et al. (2003) and Yunus et al. (2023) [73,74]. Zhu et al. reported that patchouli alcohol also has toxicity against formosan subterranean termites, *Coptotermes formosanus* Shiraki, besides having repellent properties [73]. The analysis results of the cinnamon bark essential oil aroma using the GC-MS headspace repellent method showed that the two dominant components with highest concentration were cinnamaldehyde (86.1%) with a retention time of 18,269 min (Table 6 and Figure 5). Nakasen et al. (2021) and Chansang et al. (2018) reported that *Cinnamomum verum* essential oil with major cinnamaldehyde content effectively executed 100% mosquito larvae [75,76]. Our findings confirmed the type of compounds responsible for the repellent activity of mosquitoes using headspace repellent-GC-MS methods and corroborated using LRI values analysis.

Identifying components in the patchouli and cinnamon bark essential oils must be based on the validation of LRI values, which involves comparing the experimental LRI values with reference LRI values [77]. In this study, the experimental LRI values were not close to the reference values because the standard alkane peaks generated did not provide adequate results, affecting the calculations and the retention times of alkane compounds [78]. In addition, other factors that influence the LRI values are retention time, retention factor, and relative retention. The examination of the components (constituents) of essential oils revealed several unidentified substances due to the extensive analysis and large peak numbers. In addition, there is a possibility that there exist new compounds in patchouli and cinnamon essential oils that had not been included in the library.

## 4. Materials and Methods

### 4.1. Samples

Samples used in this study were clove flowers (*Syzygium aromaticum* L.) of the Myrtaceae family, cinnamon bark (*Cinnamomum burmanii)* of the Lauraceae family, nutmeg seeds (*Myristica fragrans*) of the *Myristicaceae* family, citronella grass (*Cymbopogon nardus*) and lemongrass (*Cymbopogon citratus*) of the Poaceae family, patchouli (*Pogostemon cablin*) of the *Lamiaceae* family and turmeric (*Curcuma longa* L.) of the *Zingiberaceae* family. All samples were determined at the Research Center for the National Biology and Innovation Research Agency, Cibinong, Bogor, West Java, Indonesia with the determination result number: B-602/V/DI.05.07/3/2022.

### 4.2. Essential Oils Isolation

The steam distillation method was applied to obtain the essential oils of the cinnamon bark, clove flowers, patchouli, nutmeg seed, citronella grass, lemongrass, and turmeric rhizome. Samples consisting of cinnamon bark, clove flowers, patchouli, nutmeg, lemongrass, citronella grass, and turmeric were prepared and cleaned (wet sorting) by washing them with running water and aerated. The dried cinnamon bark, clove flowers, and nutmeg seeds were then processed into powder using a pollinator machine sifted through a 5/8 mesh (coarse powder). Patchouli, lemongrass, and citronella grass were cut into 5–10 cm pieces and the turmeric rhizome was cut into 1 cm pieces. The powder of cinnamon bark, clove flower, nutmeg, together with cut pieces of patchouli, lemongrass, citronella grass, and turmeric rhizome, was weighed to obtain a final weight of 5000 g each and then wrapped in a flannel cloth. Alternately, the steam distillation was carried out for each package by placing it in a kettle filled with water as high as the surface of the container (not far below the filter) and then heating it under low pressure for 7–10 h.

### 4.3. Activity Evaluation of Essential Oils’ Repellent Power

*Aedes aegypti* female mosquitoes were reared starting from egg-larva-pupa-imago. Eggs were soaked in water for 1–7 days and hatched into larvae. The larvae develop depending on temperature and availability of feed and form pupae in 7–9 days. However, at low temperatures, it will take several weeks for the emergence of adult mosquitoes. Therefore, in this study, the mosquitoes used were 7–24 days old. The mosquitoes were also sterile to ensure these were safe from parasites and virus infections. Upon testing, the mosquitoes were placed into three mosquito cages with each cage consisted of 25 mosquitoes. Ingredients in the form of purified essential oils of clove flowers, cinnamon, nutmeg, lemongrass, citronella grass, patchouli, and turmeric were also prepared to a concentration of 10% (5 mL of essential oil in 50 mL ethanol). The tests solutions were applied on the left arms of three volunteers (as a positive control). Then, these waited for 1 h for the ethanol to evaporate (reducing the presence of ethanol) leaving only the essential oil on the arm, while their right arms were not treated (negative control). After one hour, the volunteers’ arms were placed into the mosquito cage from 0 to 6 h. At hour 0, the right arm of each volunteer was inserted into each mosquito cage and for 5 min, the number of mosquitoes that perched were counted; then, the arm was shaken to remove the mosquitoes and to prevent them from biting and sucking the blood. In the second 5 min, the left arm of each volunteer was placed into each cage as before and the number of mosquitoes that perched on it were also counted and the arm was shaken to remove the mosquitoes and prevent them from biting and sucking the blood. The treatment was repeated every 1 h until hour 6. The engorged mosquitoes were replaced with new mosquitoes for every treatment. 

The percentage of repelling power of the essential oils against the mosquitoes was then calculated using the following formula:

$$\mathrm{RP}\;\left(\%\right)=\frac{C-P}{C}\times 100\;\%$$

*C* = number of mosquitoes perched on the control arm.

*P* = number of mosquitoes perched on the treatment arm. 

RP = Repellent power against mosquitoes.

### 4.4. Analysis of Essential Oil Aromas’ Components Using Gas Chromatography–Mass Spectrometry (GC-MS)

A total of 1 μL of each essential oil was injected into Agilent 7890A Injector and Agilent 5977B GC/MS Detector. The column used was a DB-5-MS (5%-phenyl)-methylpolysiloxane with a length of 30 m and a diameter of 0.25 mm, with a total running time of 60.00 min. The carrier gas used was helium, with an interface temperature of 210 °C and an oven temperature ramped from 60 °C to 246 °C at a rate of 3 °C/min. After 2 min, it was ramped again at a rate of 5 °C/min until reaching 200 °C. The split ratio was 1:20, with a gas flow of 1 mL/min. The auxiliary temperature was 250 °C, and the MS acquisition mode was scan, with a solvent delay of 3 min. The low mass was 40 m/z, the high mass was 500 m/z, the MS source was set at 230 °C, and the MS quadrupole was set at 150 °C.

The retention time of reference alkanes (C_8_–C_40_) injected into the GC-MS under the same circumstances and column as the sample analytes was used to calculate the LRI value. The retention time of the analyte was compared with the alkane homologs. Although the temperature differed, the retention index (LRIx) values obtained from this comparison were similar to the values obtained in the earlier study, which also used the alkane homologs as reference compounds and a similar column for the analysis of the investigated components. 

The equation used to calculate the LRI value is as follows.

$$LRI_{x}=\left\{(\frac{t_{x}-t_{n}}{t_{n+1}-t_{n}})+n\right\}\times 100$$

### 4.5. Equipment Development

The headspace repellent equipment consisted of two inhalators made of acrylic material with a size of 10 × 10 cm^3^. The tool was equipped with a water bath with a temperature of 50 °C and vial size of 50 mL containing 10% concentration of essential oil. In addition, the tool was also equipped with a hose as a connector to the inhalators (Figure 1). 

### 4.6. Analysis of Essential Oils Activities Test Using Headspace Repellent Method 

In total, 25 *Aedes Aegypti* adult female mosquitoes were placed in the inhalator to perform the repellency test. Each of the essential oil with a concentration of 10% was added into the vial and placed on top of the water batch. The headspace repellent was then turned on with a batch of water with a temperature of around 50 °C. The aroma of essential oil flowed into the inhalator containing the mosquitoes for 5 min and the number of mosquitoes that moved to the air-free inhalator was then counted. The observation was conducted three times with repetitions. Each trial was replaced with a new inhalator.

### 4.7. Analysis of Essential Oils Aromas’ Components Using GC-MS Headspace Repellent

The assembled headspace repellent equipment was prepared and 25 *Aedes aegypti* adult female mosquitoes were placed into the inhalator to perform the repellency test. Furthermore, an amount of 5 mL essential oil was added into the vial and then placed on top of the water batch. The assembled headspace repellent was then turned on by pressing the on/off button and the temperature set at around 50 °C. The aroma of essential oil flowed into an airtight inhalator to observe the movement of mosquitoes. The aroma was taken using a 1 μL injection syringe and then injecting it into the GC-MS equipment. 

### 4.8. Statistical Analysis

The results of the repellency test were analyzed statistically using a variant with a significance level (α = 0.05%) utilizing the software SPSS (Statistical Product and Service Solution) version 25. The Kolmogorov–Smirnov test results showed that the data was normally distributed with a significance value >0.05. This stage was then followed by a homogeneity test which resulted in a value of 0.068 > 0.05 and an ANOVA test which resulted in a significance value of 0.000 < 0.05.

## 5. Conclusions

The distillation results of clove flowers (*Syzygium aromaticum* L.), cinnamon bark (*Cinnamomum burmanii*.), nutmeg seeds (*Myristica fragrans*), citronella grass (*Cymbopogon nardus*), lemongrass (*Cymbopogon citratus*), patchouli (*Pogostemon cablin*), turmeric (*Curcuma longa* L.) produce different colors and yield percentages. 

The essential oil repellent test against *Aedes aegypti* mosquitoes showed that the essential oil concentration of 10% gives an average repellent power of 95.2% (patchouli), 94.7% (cinnamon), 85% (nutmeg), 83.8% (turmeric), 80.4% (clove flowers), 72.2% (citronella grass), and 71.4% (lemongrass) essential oils. 

Meanwhile, the patchouli essential oil components using the GC-MS method identified 26 components in the aroma where the dominant components were patchouli alcohol (42.7%), azulene (10.8%), alpha-guaiene (9.22%), and seychellene (8.19%), whereas for cinnamon essential oil, five components were identified, with cinnamaldehyde (90.4%) being the highest concentration of component. 

Patchouli alcohol in *Pogostemon cablin* and cinnamaldehyde in *Cinnamomum burmanii* are found to be the major component using headspace repellent equipment. Hence, it is predicted as the compound responsible for the repellent activity against *Aedes aegypti* mosquitoes.

## Figures and Tables

**Table 1:** Average repellent power of essential oils.

Essential Oils	Repellent Power at Hour (%)							
	0	1	2	3	4	5	6	Average
Patchouli	98.5 ± 0.1	95.8 ± 3.4	96.3 ± 2.8	96.3 ± 0.5	94.8 ± 2.5	91.2 ± 3.1	93.2 ± 1	95.1 ± 2.19
Cinnamon bark	100 ± 0	99.6 ± 0.57	96.7 ± 2.3	90.6 ± 4.0	90.5 ± 7.5	93.7 ± 2	92 ± 1.5	94.7 ± 3.75
Nutmeg	97.5 ± 1.7	93.8 ± 0.57	86.3 ± 1.52	83.5 ± 6.80	74.5 ± 8.38	71.1 ± 5.56	88.6 ± 7.81	85 ± 8.89
Turmeric	89.8 ± 22.1	84.3 ± 4.0	82.3 ± 10.9	81.1 ± 3.5	85.2 ± 2.0	81.8 ± 16.8	82 ± 10.8	83.8 ± 2.8
Clove flowers	100 ± 0	98.4 ± 2	78.7 ± 3.6	77.5 ± 4	69 ± 6.4	72.3 ± 23.8	66.7 ± 22.9	80.4 ± 12.5
Citronella grass	98.7 ± 1.5	65.7 ± 26.8	67.7 ± 19.2	63.5 ± 6.5	70.5 ± 13.6	69.8 ± 12.7	69.1 ± 4.5	72.2 ± 11
Lemongrass	100 ± 0	93.5 ± 6.0	78.7 ± 13.8	59.3 ± 31.4	63.4 ± 11.0	59.4 ± 10.5	46 ± 9.6	71.4 ± 18.3

±SD (standard deviation).

**Table 2:** Activity test of the essential oils aroma using headspace repellent method.

Essential Oils	Repellent Power (%)			
	1	2	3	Average
Patchouli	96 ± 1.0	92 ± 2.0	96 ± 2.0	96 ± 2.0
Cinnamon bark	100 ± 0.1	92 ± 2.0	92 ± 2.0	94 ± 1.7

±SD (Standard Deviation).

**Table 3:** Analysis of patchouli essentials oil aroma components using GC-MS Method.

Peak	RT	LRI Exp	LRI Ref	Component	Area %	% Concentration
1.	5.30	1004	1011 [40]	3-Carene	272	1.05
2.	6.34	1018	1031 [41]	beta-Phellandrene	162	0.63
3.	6.49	1019	1032 [42]	Cyclohexene 4-methylene-1-(1-methylethyl)	102	0.39
4.	22.44	1337	1380 [41]	beta-patchoulene	454	1.76
5.	22.70	1383	1392 [43]	Cyclohexane. 1-ethenyl-1-methyl-2,4-bis(1-methylethenyl)-, [1S-(1α,2β,4β)]	244	0.95
6.	23.91	1422	1428 [44]	Beta-Caryophyllene	329	1.28
7.	24.75	1452	1439 [41]	α-Guaiene	2368	9.22
8.	25.26	1458	1446 [40]	Seychellene	2104	8.19
9.	25.71	1462	1443 [40]	8-β-cedrane	1165	4.53
10.	25.79	1469	1431 [40]	β -Gurjunene	223	0.86
11.	27.05	1497	1501 [40]	Aciphyllene	449	1.74
12.	27.45	1512	1505 [45]	Azulene. 1,2,3,5,6,7,8,8a-octahydro-1,4-dimethyl-7-(1-methylethenyl)-, [1S-(1α,7α,8aβ)]	2795	10.8
13.	30.00	1591	-	n.d	178	0.69
14.	30.11	1632	-	n.d	452	1.76
15.	30.47	1549	1582 [40]	Caryophyllene oxide	434	1.69
16.	30.60	1598	-	1,1,4,7-Tetramethyldecahydro-1H-cyclopropa[e]azulene-4,7-diol	199	0.77
17.	31.48	1424	-	n.d	108	0.42
18.	31.97	1055	-	n.d	176	0.68
19.	32.34	1628	-	n.d	334	1.3
20.	32.79	1655	-	n.d	147	0.57
21.	34.22	1668	1658 [40]	Patchouli alcohol	10,000	42.7
22.	34.69	1695	-	n.d	102	0.39
23.	34.80	1789	-	n.d	270	1.05
24.	35.03	1379	-	n.d	264	1.02
25.	35.63	1652	-	4-Hydroxy-6-methyl-3-(4-methylpentanoyl)-2H-pyran-2-one	639	2.48
26.	41.52	1867	-	(E)-Atlantone	105	0.4

LRI exp = Experimental LRI calculation results on column DB-5. n.d. = no detection.

**Table 4:** Analysis of cinnamon bark’s essentials oil components using GC-MS Method.

Peak	RT	LRI Exp	LRI Ref [40]	Component	% Concentration
1	19.26	1206	1270	E-Cinnamaldehyde	73.0
2	19.84	1220	1259	3-Phenyl-2-Propen-1-ol	0.96
3	21.46	1328	1356	Eugenol	1.22
4	25.02	1408	1429	Coumarin	1.51
5	25.46	1410	1440	Z-Cinnamyl acetate	5.85

LRI exp = Experimental LRI calculation results on column DB-5.

**Table 5:** Analysis of the patchouli essential oil aroma using GC-MS headspace repellent method.

Peak	RT	LRI Exp	Area %	% Conc.	Component
1	24.51	1449	994	5.2	α-Guaiene:
2	25.03	1440	1005	5.2	Seychellene
3	27.21	1501	1371	5.2	Azulene, 1,2,3,5,6,7,8,8a-octahydro-1,4-dimethyl-7-(1-methylethenyl)-, [1S-(1α,7α,8aβ)]
4	33.77	1640	10,000	52.5	Patchouli alcohol

LRI exp = Experimental LRI calculation results on column DB-5.

**Table 6:** Analysis of the cinnamon bark essential oil aroma using GC-MS headspace repellent method.

Peak	RT	LRI Exp	Area %	% Concentration	Component
3	18.26	1190	10,000	86.1	Cinnamaldehyde
5	25.17	1389	622	5.3	Z-Cinnamyl acetate

LRI exp = Experimental LRI calculation results on column DB-5.

## Data Availability

Not applicable.

## Sample Availability

Stored in a laboratory.

## Abbreviations

LRIx:	Linear retention index of component x that is investigated
tx:	Retention time of component x (min)
t_n_:	Retention time of standard alkane with n carbon atoms eluted before the retention time of component x
t_n+1_:	Retention time of standard alkane with n+1 carbon atoms eluted before the retention time of component x
n:	Number of carbon atoms in the standard alkane eluted before component x
